# A lossless reference-free sequence compression algorithm leveraging grammatical, statistical, and substitution rules

**DOI:** 10.1093/bfgp/elae050

**Published:** 2025-01-08

**Authors:** Subhankar Roy, Dilip Kumar Maity, Anirban Mukhopadhyay

**Affiliations:** Department of Computer Science & Engineering, Academy of Technology, Adisaptagram, Hooghly-712121, India; Department of Computer Science & Engineering, University of Kalyani, Kalyani-741235, India; Department of Computer Science & Engineering, Academy of Technology, Adisaptagram, Hooghly-712121, India; Department of Computer Science & Engineering, University of Kalyani, Kalyani-741235, India

**Keywords:** Reference-free Lossless Compression, DNA and RNA Sequence Compression, FASTA, Grammar Rules, Statistical Rules, Substitution Rules

## Abstract

Deoxyribonucleic acid (DNA) or ribonucleic acid (RNA) sequence compressors for novel species frequently face challenges when processing wide-scale raw, FASTA, or multi-FASTA structured data. For years, molecular sequence databases have favored the widely used general-purpose Gzip and Zstd compressors. The absence of sequence-specific characteristics in these encoders results in subpar performance, and their use depends on time-consuming parameter adjustments. To address these limitations, in this article, we propose a reference-free, lossless sequence compressor called GraSS (Grammatical, Statistical, and Substitution Rule-Based). GraSS compresses sequences more effectively by taking advantage of certain characteristics seen in DNA and RNA sequences. It supports various formats, including raw, FASTA, and multi-FASTA, commonly found in GenBank DNA and RNA files. We evaluate GraSS’s performance using ten benchmark DNA sequences with reduced number of repeats, two highly repetitive RNA sequences, and fifteen raw DNA sequences. Test results indicate that the weighted average compression ratios (WACR) for DNA and RNA sequences are 4.5 and 19.6, respectively. Additionally, the entire DNA sequence corpus has a total compression time (TCT) of 246.8 seconds (s). These results demonstrate that the proposed compression method performs better than several advanced algorithms specifically designed to handle various levels of sequence redundancy. The decompression times, memory usage, and CPU usage are also very competitive. **Contact:**  anirban@klyuniv.ac.in

## Introduction

The ongoing advancements in Next Generation Sequencing (NGS) technology [1] have resulted in the rapid accumulation of sequences from new species [2, 3]. This necessitates the development of a reference-free compression method with high compression ratios, speed, and memory efficiency [4, 5]. The concept of reference-free sequence compression was first proposed in 1986 [6], and the first practical method, *biocompress*, was developed in 1993 [4]. However, considering its 31-year lifespan, it is evident that a more effective compressor is needed as sequencing costs continue to decrease, despite Moore’s Law being broken [3]. The exponential rise of genomic sequences over time has raised the expense of storage and the burdens associated with transmission [7–9].

Several benchmark lossless text compression algorithms are widely used in practice. These include Zstd [10], Gzip [11], Bzip2 [12], and 7-zip [13]. These algorithms have proven their effectiveness in various applications and are commonly employed for compressing text-based data, including genome sequences. The benchmark lossless text compression algorithms excel in languages with a wide alphabet, like English. However, when it comes to biological sequences such as deoxyribonucleic acid (DNA), these algorithms may not perform optimally. This is due to the fact that DNA sequences usually contain the specific letters adenine (A), cytosine (C), guanine (G), and thymine (T), as well as other special characters, and they have unique biological characteristics, including repeats, palindromes, and minuscule alphabets [14]. However, utilizing the training mode with a dictionary in the Zstd [10] compressor significantly enhances the compression ratio attainable on small datasets.

In the field of sequence compression, specialized algorithms can be categorized into two main groups: reference-free and reference-based algorithms [15], and implementations in two other groups: sequential and random-access [16, 17]. Reference-free algorithms [18] leverage the intrinsic characteristics of the sequences to be compressed, while reference-based algorithms [19] rely on a reference sequence for compression. Reference-free compression methods analyze the target sequence ahead of time to find patterns such as exact or nearly exact duplicates, palindromes, and other recurring structures. They then use these patterns in algorithmic changes, for example, by using indices to encode the sequence, enabling efficient compression. In contrast, reference-based techniques compare the target sequence with a reference sequence [20]. They encode the differences between the two sequences, achieving high compression ratios. Reference-free compression may yield advantageous compression ratios (CR) for novel species that may lack adequate references [21, 22]. They speed up processing more than reference-based compression since they do not require reference sequence preprocessing [22]. However, even in the absence of a proper reference sequence, reference-free compression can still be effective. Choosing the appropriate reference sequence for a new species can be a time-consuming task. However, once a suitable reference is established, reference-based compression techniques can be employed, offering potentially higher compression ratios.

Compression algorithms can be classified into two categories based on their ability to preserve information: lossy and lossless compression. Lossy compression is commonly applied to images, audio, and video files where the removal of redundant or insignificant data has minimal impact on their quality [23]. On the other hand, lossless techniques ensure complete data recovery after decompression, making them vital for preserving the integrity of the compressed data. In the context of genome compression, lossless techniques are particularly important since they ensure that no data is lost during the compression process. For proper data preservation utilizing DNA media, sequence analysis [24, 25], and interpretation, this is essential. Any loss of information in this domain could potentially lead to erroneous conclusions.

In the field of bioinformatics [26], there are various compression techniques, algorithms, and software tools available [27]. Despite ongoing research and development, many databases still rely heavily on the general-purpose lossless compression techniques Zstd and Gzip. Although specialized compression algorithms have been created in an effort to replace Zstd or Gzip throughout the years, there is still opportunity for development in this area in terms of higher compression ratios, quicker processing times, and more effective CPU and memory usage.

The majority of the specialists experience one or more of the following deficiencies [27]: (i) Choices made regarding parameters, i.e., varying levels and/or threads, have a significant impact on the effectiveness of the majority of practical benchmarking procedures. (ii) As the size of the data goes beyond (>10 MB), certain benchmark methods experience significant slowdowns. When the data size exceeds 245 MB, certain algorithms are unable to handle them effectively. (iii) Some algorithms only accept [ACGTN], while they classify all other characters as ‘N’ or ‘.’. Certain algorithms do not accommodate identifiers, all IUPAC codes, lowercase letters, special characters, line length, and block length. Hence, there is a requirement for specialized algorithms that can more effectively manage these biological sequences.

The aim of our research is to devise a technique for compressing DNA and RNA sequences in commonly utilized forms, such as FASTA and raw formats, without requiring reference data. Because of its simplicity and ease of interpretation for researchers, the FASTA format is frequently used in GenBank. Owing to the significance of FASTA, an encryption tool [28] is provided. Sequences with less repetition and sequences with different degrees of intra-sequence similarity perform equally well using GraSS. GraSS utilizes a two-phase technique to achieve lossless sequence compression. The first phase separates raw sequences, including N, from auxiliary data. The second phase involves the actual compression process, which leverages grammatical, statistical, and substitution rules. The fact that GraSS is unaffected by the parameter configuration is one of its noteworthy advantages. This characteristic makes it remarkably user-friendly and straightforward to use. GraSS is versatile in its applicability, accommodating both low-repetitive and high-repetitive sequences across a wide range of sizes.

The CR of the proposed technique varies between 4.07 and 4.63, with a WACR of 4.5 when applied to ten benchmark DNA sequences that have fewer repetitions. The WACR for fifteen raw DNA sequences is 4.33, with a range of 3.78 to 4.5. The compression ratio for highly repeated RNA sequences ranges from 17.33 to 25.72, with a WACR of 19.6, surpassing most benchmark compression techniques.

The remainder of the article is organized in the following sections: Section 2 describes the background literature. Section 3 discusses the proposed approach in detail. In Section 4, we describe the data sets and machine configuration. Section 5, presents the results and findings, followed by a discussion on them. Finally, Section 6 concludes the article.

## Related works

In the field of genomic sequence compression, as of 2024, there were many specialized raw and FASTA/Q-structured sequence compressors in use. We have selected thirteen state-of-the-art compressors from the available options. The other eleven compressors are specifically made to compress DNA sequences, whereas Gzip and Zstd are common general-purpose compressors that data centers may use to compress genetic material.

The industry standard compressor, Gzip, is a flexible, cross-platform compressor that frequently compresses a variety of data types, including DNA patterns, to make them easier to access. It uses the Lempel-Ziv method and Huffman compression. Higher compression rates are what the Zstd algorithm tries to achieve. The time it takes to decompress is very short. It has a very fast entropy step, thanks to Huff0 and the Finite State Entropy (FSE) package. Even though Zstd cannot compress files as quickly, it can offer better compression ratios. It has better compression ratios for sequences that repeat a lot, but not for sequences that repeat a little.

The FASTA compressor DNA-COMPACT (DCom) [29] employs a two-pass lossless methodology that integrates contextual modeling with pattern recognition. It can function as either a reference-based or reference-free algorithm. The integration of complementary contextual models is emphasized to enhance compression performance. The speed significantly decreases when data sizes exceed 10 MB. The Unstable Huffman Tree (UHT) [30] algorithm was formulated using a greedy Huffman tree methodology, leading to substantial enhancements in compression relative to Bzip2 and Gzip. However, it fails for files sized 245 MB or above. The extension of UHT is referred to as the unstable Huffman tree without greed (NUHT) [31]. However, the inherent constraints of the NUHT compressor require a significant amount of RAM, making it unsuitable for compressing large genomes like the 13.4 GB Picea abies plant genome.

Making use of FASTQ compressors with FASTA data is simple. FQZComp [32] breaks FASTQ data; it encodes each stream separately and simultaneously, using context models and an arithmetic coder. The FASTQ must have ACGTN in it. A ‘N’ or ‘.’ stands for anything else. To run on multiple threads, DSRC 2 [33] uses Boost tools. A single thread reads the incoming FASTQ file in blocks, creating an output queue. Next, the blocks undergo multithreading, resulting in the creation of another output queue. In the final step, a single thread writes the compressed block to a file. A preprocessing method first compresses each stream in LFQC [34]. Then, a regular data compressor further compresses the stream. Minicom [22] shrinks FASTQ files using two main ideas: indexing reads with minimizers and overlapping suffixes and prefixes between two contigs. Large k-minimizers are used to index reads and divide them into smaller groups.

While Jarvis [35] is only relevant for reference-free, the GeCo [36], GeCo2 [37], and GeCo3 [38] algorithms are applicable to both referential and reference-free. Their high compression ratio makes them ideal for long-term storage and analysis [39–41]. During the computation process, the BIND algorithm [42] segregates and saves identifiers, small cases (a, c, g, t), other characters, and lengths for multi-FASTA files. The BIND method simplifies the sequences to ACGT symbols.

Despite the development of specific compression algorithms, there is still room for improvement in terms of achieving greater compression ratios, faster processing times, more efficient memory management, and CPU usage in this field. The parameter choices have a substantial impact on the effectiveness of most practical benchmarking processes. The technique is complex and requires a significant amount of time because of the parameter configuration. As the data size approaches the gigabyte (GB) region, several benchmark methods experience significant slowdowns or become incapable of handling the data. Consequently, there is a constant requirement for specialized algorithms capable of managing a diverse range of sequence sizes.

## Methodology

**Figure 1:**
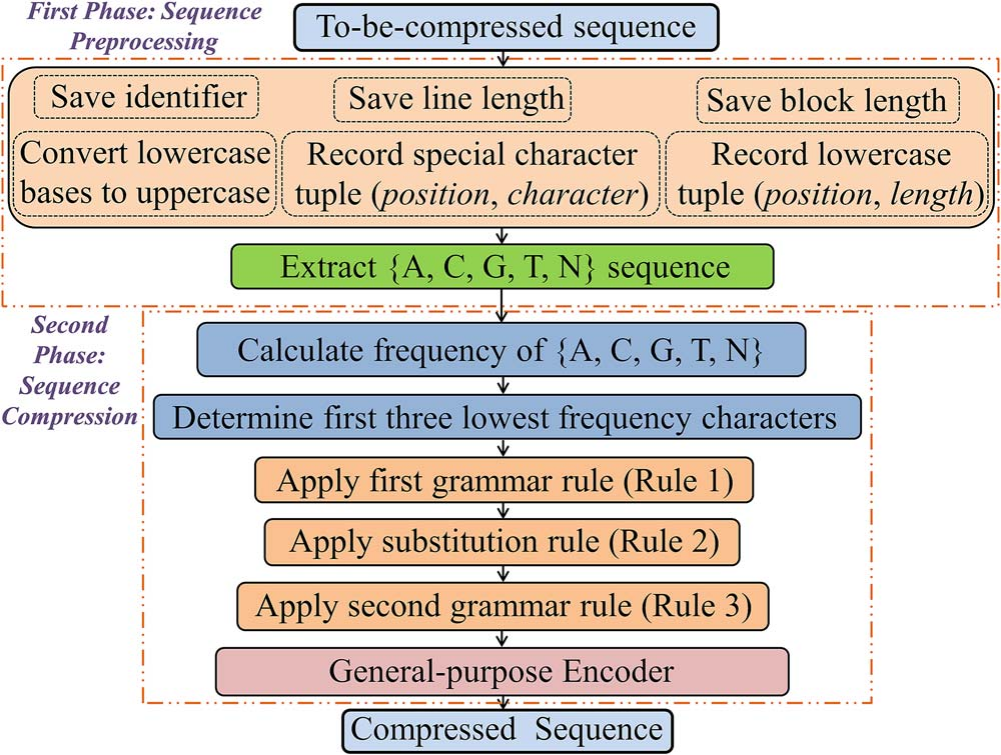
Overview of the GraSS algorithm. Sequence pretreatment and the encoding phase are its two phases. The first stage involves saving the identifier, line length, block length, lowercase bases being changed to uppercase, recording lowercases as (*position*, *length*), and recording special characters as (*position*, *character*), respectively. The first three less frequent characters are calculated during the encoding step. It then applies three coding rules after that. The BSC encoder is used to create the compressed file in its final form.

The GraSS compression algorithm is based on statistical, substitutional, and grammatical principles. It comprises six major steps divided into two separate phases. Phase one involves sequence extraction; Phase two involves utilizing a general-purpose compressor (BSC), grammar rules 1 and 2, statistical, and the substitution rule. All IUPAC codes found in the input data, including A, C, G, T, U, R, Y, S, W, K, M, B, D, H, V, and N, are supported. The symbols can be written in both lower- and upper-case, although upper-case is used before compression sequences. In the first phase, the sequence identifier, the lower-case tuple (*position*, *length*), special character tuple (*position*, *character*), line length (for FASTA/multi-FASTA), and block length (for multi-FASTA) are extracted from the genomic sequence. Since the other special characters are rare, the statistical model examines the frequency of the letters A, C, G, T/U, and N in the sequence. Genome datasets are taken into account in all of the examples, figures, and algorithms. For RNA sequences, the same process applies. The grammar rule reduces the character set from “A, C, G, T, N” to “A, C, Z”, “A, G, Z”, “A, T, Z”, “C, G, Z”, “C, T, Z”, or “G, T, Z”, effectively reducing it from 5 to 3 characters. In the substitution rule, three characters are replaced with 0, 1, and 2, respectively. The second grammar rule further reduces the character set by half. GraSS supports both the genomic raw sequence format and FASTA/multi-FASTA data. Figure 1 illustrates the basic flow of GraSS using a block diagram. Any general-purpose encoder can be used in the last stage. However, we used a BSC [43] compressor, as it is parameter-independent. The following subsections provide a detailed workflow for each stage. The decompression process of GraSS is briefly introduced in Section 3.3.

### First phase

The initial stage involves preprocessing the given input sequence. This process includes retrieving the sequence identification and determining the line length and block length for FASTA/multi-FASTA data. The line length is encoded using run-length encoding (RLE). The resulting data is then saved in a separate file named F2, along with the identification. Before storing the data in file F1, lowercase characters are converted to uppercase characters. The position and length tuples are stored in the same file, F2. Using static entropy encoding and delta coding, special characters and locations are encoded and saved in the same file, F2, respectively. Static entropy coding is utilized in order to encode the length. Additionally, a modified delta encoding technique is used to encode the position values. In the case of sequence blocks, their lengths are encoded and stored in F2 using modified delta coding. Assume that the lengths of the sequence (without the identifier) and other special characters are denoted as $n_{seq}$, $n_{spl}$, and the preprocessed sequence is denoted as $n_{tar}$, accordingly. The relationship described below is valid as a result.


\begin{align*}& n_{tar} = n_{seq}-n_{spl} \end{align*}

Algorithm 1 is used to describe the specifics of this stage. *Example*  1 showed the results for a sample to-be-compressed sequence from this step.




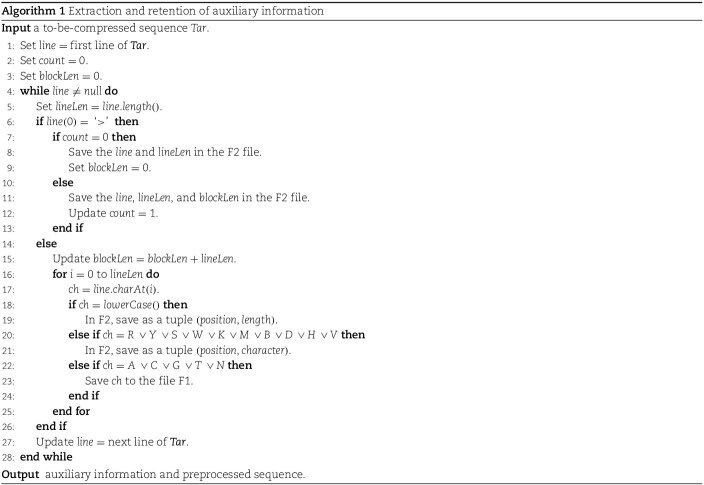

Example 1. seq1.fa

>chr

ATTGCATGTcgatggATGGggaAAA

ATCGataggatAGATTTTTAAAACC

CNNNNYYY

The following are included in the preprocessed output from file seq1.fa:

Sequence identifier (F2): >chr;

Lower case tuple (F2): (9, 6), (4, 3), and (7, 7);

Special character tuple (F2): (55, 24), (1, 24), and (1, 24);

Line length (F2): 25;

Block length (F2): 58;

Sequence (F1): ATTGCATGTCGATGGATGGGGAAAAATCGATAGGA

TAGATTTTTAAAACCCNNNN

### Second phase

The second phase of the process involves working with the raw sequence obtained from the first phase. The specific methodology used in this phase is described in *Example*  2. To effectively model the statistics of the sequence, it is necessary to determine the frequencies of the nucleotides A, C, G, T, and N in file F1. In order to reduce the character set from 5 to 3, the phase identifies the characters with the smallest to third-smallest frequencies in file F1. A flag character, Z, is introduced to facilitate the application of first-level grammar rules such as N $\rightarrow $ ZZ, G $\rightarrow $ ZT, C $\rightarrow $ ZA. These three selected characters are then encoded using a replacement method, representing them as 0, 1, and 2. As a result, the sequence will consist of 0s, 1s, and 2s. The substitution rule requires little time and physical memory in exchange for providing a unique second grammatical rule. Applying the second-level grammar rule, the sequence is halved in length compared to its original size. This grammar utilizes the following rules: 00 $\rightarrow $ P, 01 $\rightarrow $ Q, 10 $\rightarrow $ R, 02 $\rightarrow $ S, 20 $\rightarrow $ U, 11 $\rightarrow $ V, 12 $\rightarrow $ W, 21 $\rightarrow $ X, 22 $\rightarrow $ Y. Files F2 and F3 are encoded using the block-sorting encoder technique, often known as BSC, after the nine-character stream file has been stored in file F3. Algorithm 2 explains the specifics of **Steps 1** and **2**. The details of **Steps 3** through **7** are described in detail in Algorithm 3.




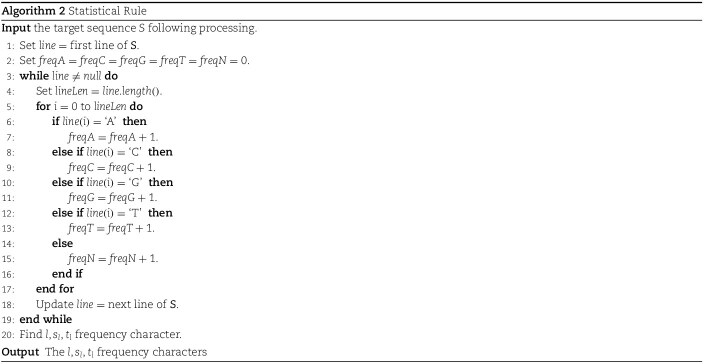






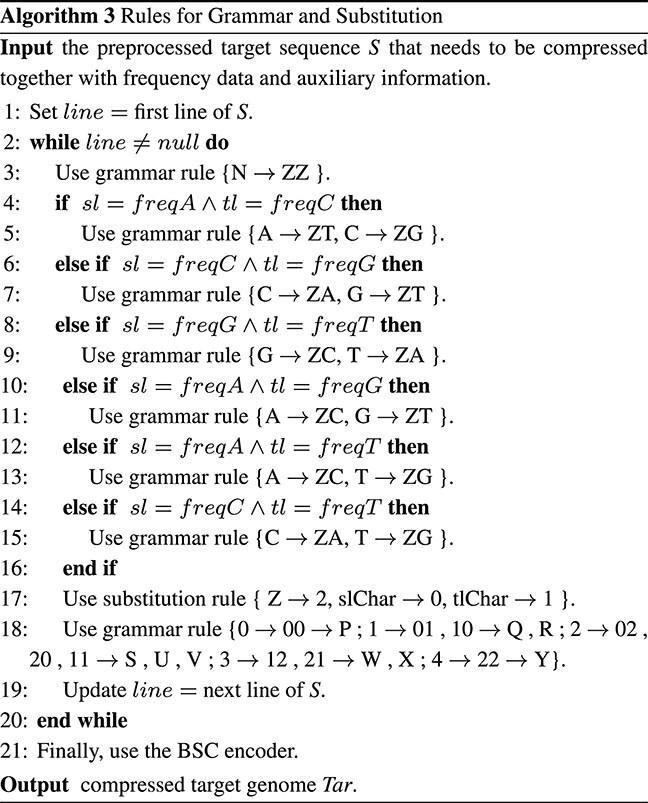



Let $l$, $s_{l}$, and $t_{l}$ represent the frequencies of the three characters with the lowest frequencies. The two remaining characters have a combined frequency of $o_{l}$. As a result, the relationship $n_{tar} = l+ s_{l} + t_{l} + o_{l}$ is true. The first grammar and substitution model gives the number of characters as $\tilde{n}_{tar} = 2 \times (l+s_{l}+t_{l})+o_{l}$. The second grammar model calculates character count as $\hat{n}_{tar} = \tilde{n}_{tar} \div 9$.

Example 2. *S* = ATTGCATGTCGATGGATGGGGAAAAATCGATAGG ATAGATTTTTAAAACCCNNNN For a certain target genome FASTA/multi-FASTA file, the detailed steps for second phase encoding is as follows:

Step 1: Calculating the frequency of appearance of the letters A, C, G, T, and N in the previously stated sequence S: *Freq*(A) = 50 *Freq*(C) = 14 *Freq*(G) = 10 *Freq*(T) = 80 *Freq*(N) = 4
Step 2: Determining the first three letters that appear the least frequently: Small(*Freq*) = *Freq*(N) = 4 SndSmall(*Freq*) = *Freq*(G) = 10 TrdSmall(*Freq*) = *Freq*(C) = 14
Step 3: The least frequent character is changed to “ZZ”, the next least frequent character is changed to the highest frequency symbol (HFS), and the third least frequent character is changed to the second HFS preceded with the letter Z. The first grammatical principle is as follows: Rule 1 = {N $\rightarrow $ ZZ, G $\rightarrow $ ZT, C $\rightarrow $ ZA} Following replacement, the sequence becomes, ATTZTZAATZTTZAZTATZTZTATZTZTZTZTAAAAATZAZTATAAT AZTATTTTTAAAAZAZAZAZZZZZZZZ $\ldots $
  The additional characters introduced in this stage total 28, increasing the overall character count from 158 to 186.
Step 4: Substituting 0, 1, and 2 for the next three characters. The substitution guideline is as follows: Rule 2 = {T $\rightarrow $ 0, A $\rightarrow $ 1, Z $\rightarrow $ 2}
  Now, the pattern of the sequence becomes 1002021102002120102020102020202011111021201011012010000011 11121212122222222$\ldots $
Step 5: To replace two consecutive integers, use the language shown below. The following is the second grammatical principle: Rule 3 = {0 $\rightarrow $ 00 $\rightarrow $ P ; 1 $\rightarrow $ 01, 10 $\rightarrow $ Q, R ; 2 $\rightarrow $ 02, 20, 11 $\rightarrow $ S, U, V ; 3 $\rightarrow $ 12, 21 $\rightarrow $ W, X ; 4 $\rightarrow $ 22 $\rightarrow $ Y}
  The sequence will be as follows after applying the aforementioned rules: RSSVSPXURUURUUUUVVRXURVQURPPVVW WWYYYY$\ldots $
Step 6: The last phase uses block-sorting encoding (BSC) to encode the intermediate data.

### Decompression

During the decoding process, the inverse operations of encoding are performed. The compressed file is first processed and decompressed by the BSC decompressor. Then, in reverse order, the second grammar rule, the substitution rule, and the first grammar rule are applied to reconstruct the original sequence. The sequence identifications (IDs) are restored in their original form and recorded in the decompressed file. Next, the block length is decoded using modified delta coding, while the line length is decoded using reverse RLE. Using delta coding and static entropy coding, the special character locations and the individual characters are decoded. Subsequently, the positions of lowercase letters are decoded using modified delta coding, and the lengths of lowercase segments are decoded using static entropy encoding. Finally, the lowercase letters are converted back to uppercase, completing the decoding process.

## Data Sets and Configuration of the Machine

### Data formats

Genome sequence data is available in a variety of formats [21]. We adopted raw sequence [44], FASTA, and multi-FASTA formats in this work because they are well-known in Gen-Bank and easy for researchers to read and analyze. FASTA format is widely used in many databases, including diverse projects [27, 45, 46], and has become the norm in genomic investigations. Given its widespread adoption, the FASTA format will continue to play a crucial role in storing and exchanging genetic data in the field of genomics.

### Used data sets

In order to assess the performance of GraSS, we have employed three distinct categories of datasets. Initially, we conducted studies on 10 standard DNA sequences with lengths ranging from 50 KB to 984246 KB and a lower degree of repetitiveness. These datasets contain the genetic information of many organisms, such as algae, mammals, bacteria, fungus, protists, and viruses [47, 48]. They provide a comprehensive analysis of genetic information that is less repetitive. We utilized extremely repetitive RNA sequences called “*SILVA_132_LSURef*” and “*SILVA_132_SSURef_Nr99*” [49], which had sizes of 595993 KB and 1083003 KB, respectively. Ultimately, we have employed a DNA corpus (509 MB) that exhibits minimal inter-similarity [39, 44, 50]. We have included sources to provide additional supporting data in the reference [50, 51]. The benchmark data is fully described in Supplementary File S1

### Computing machine

All trials were conducted on an Amazon AWS cloud computer, which operated on Ubuntu 18.04.1 LTS (64-bit). The computer was equipped with two powerful Intel Xeon CPUs (E5-2643 v3 @ 3.4 GHz, 6 cores) and 128 GB of RAM. To compile and execute the program, JDK 18 was utilized.

## Results and discussion

In order to compress specified DNA and RNA that are less and more repetitive, we propose a customized compression approach that is lossless and does not require a reference, resulting in efficient compression. In our study, we assessed the performance of GraSS in comparison to 13 state-of-the-art methods: Gzip, Zstd, DCom [29], FQZComp [32], DSRC 2 [33], LFQC [34], UHT [30], NUHT [31], Minicom [22], GeCo [36], Jarvis [35], GeCo2 [37], and GeCo3 [38]. We operate each compressor using the best possible parameter configuration to achieve the highest compression ratio, as specified in the original articles. Supplementary Files 2, 3, and 4 contain the specific information.

The compressed file sizes produced by GraSS and nine state-of-the-art algorithms (Gzip, Zstd, DCom, FQZComp, DSRC 2, LFQC, UHT, NUHT, and Minicom) on 10 DNA sequences with reasonably low repetition can be found in Supplementary File S2. Among the ten datasets, our proposed technique performs better than all others in one case and ranks second in six cases. The performance of the remaining three datasets is highly competitive with that of the top-performing LFQC. The compressed size of two highly repetitive RNA sequences can be seen in Supplementary File S3. Moreover, it has been observed that the proposed approach surpasses Gzip, FQZComp, DSRC 2, UHT, NUHT, and Minicom in terms of compression performance. GraSS’s distinctive design, which does not rely on exact or approximate repeats, palindromes, or other repeating structures within a sequence, resulted in a higher compressed file size compared to the proposed technique when utilizing Zstd. The Supplementary File S4 includes a compressed list generated by the GraSS method, as well as six other advanced algorithms: Gzip, Zstd, GeCo, Jarvis, GeCo2, and GeCo3. These algorithms were applied to a DNA sequence corpus consisting of fifteen raw sequences. The compression efficiency exceeds that of Gzip and Zstd. However, GeCo, Jarvis, GeCo2, and GeCo3 surpass GraSS. The rationale is that the proposed method is largely tailored for the FASTA format, although it can also accommodate raw data. However, the compression efficiency will not match that of the FASTA format. This exemplifies the efficacy of our approach in producing optimal compressed file sizes for diverse datasets.

GeCo, GeCo2, Jarvis, and GeCo3 were originally developed for the long-term storage of the genome primary domain [ACGT]. They demonstrate outstanding WACR, as shown in Table 2. We have assessed the proposed approach against these four compressors, as outlined in Table 2, employing portions of the same dataset utilized in their original studies. To apply them to FASTA/Q files, a conversion to the raw data is required [27, 38]. Therefore, we have not tested them against the sequences in the FASTA data presented in Table 1 of the revised manuscript. The compressors DSRC 2, LFQC, FQZComp, and Minicomare are compatible with FASTQ format, whereas DCom, UHT, and NUHT are appropriate for FASTA. To apply them to the raw data of Table 2, the researcher must convert them to the appropriate format [27]. Therefore, we have omitted them from the sequences of raw files in Table 2. However, as Gzip and Zstd are general-purpose compressors, we have evaluated their performance across several data formats.

**Table 1:** The CR, WACR, CT (s), and TCT (s), utilizing the proposed method plus nine additional compressors on ten DNA sequences and two RNA sequences

ID	Gzip		Zstd		DCom		FQZComp		DSRC 2		LFQC		UHT		NUHT		Minicom		GraSS	
GCF_001884535.1	3.3	0.15	3.61	**0.04**	**4.23**	5.1	4	1.02	3.89	0.871	2.46	0.538	3.86	2.38	3.91	1.18	1.63?	5.856	*4.07*	*0.149*
GCA_000398605.1	3.44	0.63	3.64	**0.252**	**4.34**	30.17	4.13	1.03	3.95	0.925	3.9	2.555	3.92	4.51	3.98	2.58	3.62?	5.966	*4.21*	*0.483*
GCA_000211355.2	3.49	1.21	3.76	**0.851**	**4.45**	69.98	4.34?	1.03	4.19	*1.024*	*4.4*	6.716	4.09	9.08	4.16	4.21	4.14?	6.031	**4.45**	1.141
GCA_000988165.1	3.22	5.17	3.7	3.5	4.02	239.8	4.12?	**1.04**	4.06	*1.09*	**4.32**	28.48	3.57	23.93	3.7	7.31	4.1?	6.397	*4.16*	3.261
GCA_000165345.1	3.24	8.383	3.59	6.21	4.06	391.4	4.05?	**1.19**	3.97	**1.19**	**4.23**	42.4	3.78	38.48	3.71	9.83	4.02?	6.645	*4.09*	*5.34*
GCA_000497125.1	3.24	10.76	3.78	9.7	$\ast $	$\ast $	4.04?	**1.67**	3.94	**1.67**	**4.38**	56.08	3.69	56.18	3.73	15.64	*4.16*?	9.216	4.09	*7.45*
GCA_001606155.1	3.29	20.84	3.73	20.79	$\ast $	$\ast $	4.15?	**1.651**	4.04	**1.651**	**4.36**	98.28	3.74	97.86	3.78	21.29	4.13?	12.226	*4.17*	*12.13*
GCF_000240135.3	3.24	34.13	3.56	36.49	$\ast $	$\ast $	3.99?	**1.997**	3.92	*2.46*	**4.08**	152.4	3.64	164.1	3.79	34	*3.99*?	8.079	*3.99*	*15.26*
GCA_002205965.2	3.22	240.4	4.4	458.79	$\ast $	$\ast $	4.07?	*28.89*	3.98	**6.759**	**4.65**	442.2	*#*	*#*	3.78	268.2	*4.63*?	120.9	4.28	128.89
GCF_000002235.4	3.56	508.7	4.48	1118	$\ast $	$\ast $	**5.04**?	*75.78*	4.31	**12.96**	4.86	833.8	*#*	*#*	4.06	697.8	*4.91*?	585.4	4.63	392.7
WACR & TCT (s)	3.45	830.4	4.39	1654.6	4.23	736.5	*4.73*	*115.3*	4.21	**30.6**	**4.88**	1663.4	3.69	396.5	3.97	1062	4.78	766.7	4.5	566.8
SILVA 132 LSURef	6.25	280.1	**39.07**	375.2	$\ast $	$\ast $	22.7?	*30.58*	6.48	**6.74**	*26.45*	492.4	*#*	*#*	3.96	425	26.2?	816.6	25.72	160.07
SILVA 132 SSURef Nr99	5.37	557.2	**20.03**	1089.4	$\ast $	$\ast $	15.6?	*39.76*	6.72	**10.59**	15.6	764.5	*#*	*#*	3.99	742.3	15.9?	5794	*17.33*	319.23
WACR & TCT (s)	5.65	837.3	**24.22**	1464.6	$\ast $	$\ast $	17.56	*70.3*	6.63	**17.3**	18.23	1256.9	*#*	*#*	3.98	1167.3	18.48	6610.6	*19.6*	479.3

The question mark indicates results where the decompression produces different results than the input file. The bold font signifies the best result in the row, while the italic font denotes the second-best result. $\ast $ The sequences were not tested due to a significant increase in time. *#* The sequences fail to compress. The first column of each compressor is CR, and the second column is CT. The WACR is the weighted average compression ratio, and TCT (s) is the total compression time.

**Table 2:** The CR, WACR, CT (s), and TCT (s), utilizing the proposed method plus six additional compressors on 15 DNA sequences

ID	Gzip		Zstd		GeCo		Jarvis		GeCo2		GeCo3		GraSS	
BuEb	3.25	**0.009**	3.92	*0.012*	3.97	6.05	**4.04**	0.092	**4.04**	0.129	*4.03*	0.1042	3.78	0.174
AgPh	3.37	*0.037*	3.96	**0.026**	4.04	6.12	*4.09*	0.138	**4.11**	0.05	*4.09*	0.11	3.93	0.169
YeMi	3.59	*0.094*	4.12	**0.049**	4.29	6.29	*4.37*	0.142	**4.39**	0.259	*4.37*	0.175	4.22	0.256
AeCa	3.58	2.1	3.97	*1.19*	4.13	13.28	4.18	*1.19*	*4.19*	1.37	**4.2**	4.4	4.1	**1.1**
HePy	3.67	2.21	4.02	1.38	4.37	13.36	**4.46**	*1.34*	*4.44*	1.28	4.45	7.02	4.24	**1.13**
HaHi	3.63	5.27	3.99	*2.368*	4.29	24.01	*4.32*	3.38	4.31	2.51	**4.33**	15.82	4.19	**2.356**
EsCo	3.57	5.38	4.03	2.94	4.18	26.77	**4.24**	5.78	*4.23*	*2.93*	**4.24**	19.24	4.07	**2.91**
PIFa	3.77	13.85	4.28	6.49	4.62	43.96	*4.67*	13.18	*4.67*	21.45	**4.71**	76.08	4.35	**4.785**
ScPo	3.57	13.29	3.78	8.13	4.2	54.01	*4.23*	15.48	*4.23*	25.234	**4.24**	72.24	4.08	**5.958**
EnIn	3.61	35.14	4.48	*25.04*	5.08	92.78	**5.19**	58.54	*5.11*	71.02	**5.19**	159.64	4.24	**14.112**
DrMe	3.6	36.76	4.08	*31.29*	4.29	103.89	*4.3*	39.09	*4.3*	79.52	**4.35**	230.88	4.11	**18.17**
OrSa	3.66	47.73	4.42	*45.18*	4.99	121.49	**5.12**	108.87	*5*	99.34	**5.12**	298.68	4.24	**19.695**
DaRe	3.77	77.1	4.81	*68.71*	5.43	145.92	**5.6**	127.1	5.45	114.29	*5.57*	420.35	4.5	**26.593**
GaGa	3.66	190.3	3.92	*189.1*	4.38	268.92	*4.41*	220.87	4.38	229.53	**4.43**	866.76	4.2	**66.855**
HoSa	3.73	*243.2*	4.37	250.5	4.88	373.63	*4.91*	352.58	4.88	292.04	**4.99**	1251.48	4.48	**82.543**
WACR & TCT (s)	3.69	672.5	4.25	*632.4*	4.73	1294.4	*4.78*	947.7	4.74	940.8	**4.82**	3422.9	4.33	**246.6**

The bold font signifies the best result in the row, while the italic font denotes the second-best result. The first column of each compressor is CR, and the second column is CT. The WACR is the weighted average compression ratio, and TCT (s) is the total compression time.

We compute the following metrics in the subsequent sections:

(1) The CR = size of the original file $\div $ size of the compressed file
(2) The WACR = total size of the original file $\div $ total size of the compressed file
(3) The compression ratio improvement percentage (CRIP) = [(GraSS WACR $\div $ comparable algorithm WACR) -1] $\times $ 100%
(4) Total (de)compression time (TCT/DCT) (s) required in a specific dataset.
(5) The maximum memory (MB) used during the compression and decompression processes.
(6) The percentage of a computer’s central processing unit (CPU) used by the state-of-the-art compressor.

### Performance comparison of compression ratio

The proposed method consistently performs better than the state-of-the-art Gzip and Zstd algorithms, except for one dataset (GCA_002205965.2), as indicated in Table 1. As demonstrated in Table 1, GraSS performs better than the customized state-of-the-art FQZComp in eight instances, surpasses DRSC 2 in all instances, outshines LFQC in three instances, and exceeds Minicom in six instances. With a WACR of 4.5 (Table 1), GraSS performs better than six composers among nine examined compressors. Utilizing the compressors (FQZComp and Minicom) listed in Table 1, the decompressed file frequently fails to completely correspond with the original file throughout the majority of data sets, notwithstanding the accurate size. While the LFQC compression ratio (4.88) surpasses that of GraSS, GraSS operates at a speed nearly three times faster than LFQC (Table 1). Compared to the state-of-the-art Gzip, Zstd, DCom, DSRC 2, UHT, and NUHT, GraSS achieved CRIP values of 30.43%, 2.51%, 6.38%, 6.89%, 21.95%, and 13.35%, respectively.

The proposed algorithm achieves a WACR value of 19.6 for the two highly repetitive RNA sequences (Table 1). It surpasses the results achieved by Gzip, FQZComp, DSRC 2, LFQC, NUHT, and Minicom, which are 5.65, 17.56, 6.63, 18.23, 3.98, and 18.48, respectively (Table 1). The CRIP values for GraSS are 246.9%, 11.62%, 195.63%, 7.52%, 392.46% and 6.06%, respectively. The Zstd algorithm achieves a WACR value of 24.22 for extremely repetitive sequences due to its design, surpassing that of GraSS.

The GraSS WACR value of 4.33 for the DNA raw sequence corpus is suboptimal, as it is mainly designed for FASTA, as illustrated in Table 2. However, the WACR values of GeCo, Jarvis, GeCo2, and GeCo3 are higher than GraSS, with values of 4.73, 4.78, 4.74, and 4.82, respectively. These approaches are effective for long-term storage due to their higher compression ratio, but at the expense of computing resources.

### Performance comparison of time complexity and execution time

Performance assessment also makes use of complexity and execution time. The following is a discussion and comparison of GraSS’s complexity analysis and execution time with the aforementioned state-of-the-art. Preprocessing and encoding take a linear amount of time. The statistical model’s time complexity is always $O(n_{tar})$. The time complexity is $O(\tilde{n}_{tar})$ for both the substitution model and the first grammar. The time complexity for the second grammar rule is $O(\hat{n}_{tar})$. The variable $n_{tar}$ represents the count of characters in the preprocessed sequence. Similarly, $\tilde{n}_{tar}$ denotes the count of characters after applying the first grammar or substitution rule, while $\hat{n}_{tar}$ represents the count of characters after applying the second grammar rule.

The results of three executions on the same data sets were averaged to determine the compression and decompression times. Table 1 presents a time-related comparison between GraSS and the nine state-of-the-art methods. The DSRC 2 method is the fastest, and the LFQC method is the slowest of all the methods listed. The design of the Zstd method causes the compression time to increase proportionally with the size of the input file. Despite multi-step processing, GraSS is 1.47 times faster than Gzip, 2.94 times faster than Zstd, 1.3 times faster than DCom, 2.93 times faster than LFQC, 1.87 times faster than NUHT, and 1.35 times faster than Minicom.

The proposed and seven state-of-the-art methods took 479.3 s, 837.3 s, 1464.6 s, 70.3 s, 17.3 s, 1256.9 s, 1167.3 s, and 6610.6 s, respectively, to encode two RNA sequences (Table 1). Minicom is the slowest technique due to hash table construction during compression, whereas DSRC 2 is the fastest. GraSS performs better than Gzip by 1.75 times, Zstd by 3.06 times, LFQC by 2.62 times, NUHT by 2.44 times, and Minicom by 13.79 times. In comparison to DSRC2 and FQZComp, the proposed method is much slower because of its multi-step processing.

GraSS execution time (Table 2) surpasses Gzip by a factor of 2.72, Zstd by a factor of 2.56, GeCo by a factor of 5.27, Jarvis by a factor of 3.84, GeCo2 by a factor of 3.81, and GeCo3 by a factor of 13.87, respectively. The advanced GeCo, Jarvis, GeCo2, and GeCo3 exhibit exceptional efficiency for prolonged storage applications. Nevertheless, GraSS is the fastest compressor for this dataset, making it highly efficient for frequent access as well.

**Figure 2:**
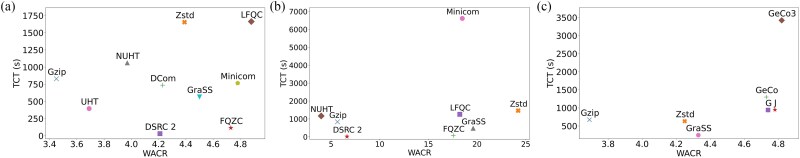
The scatter plots (a) for DNA sequences in Table 1, (b) for RNA sequences in Table 1, and (c) for sequences in Table 2 (For visual clarity, we write FQZComp as FQZC, GeCo2 as G, and Jarvis as J) demonstrate the trade-off between WACR and TCT (s).

The scatter plots (Fig. 2) illustrate the trade-off between WACR and TCT for 10 less-repetitive DNA sequences, two highly-repetitive RNA sequences, and 15 raw DNA sequences.

Supplementary File S5 contains a list of GraSS decompression timings, as well as those for the ten previously described state-of-the-art methods. The decompression time of the proposed approach is significantly greater than that of the two general-purpose algorithms, Gzip and Zstd. Because they are reversible processes, specialized algorithms take longer to complete than general-purpose algorithms. The proposed technique performs better than DCom, LFQC, and NUHT and the most advanced GeCo, Jarvis, GeCo2, and GeCo3 in terms of decomposition time. This further demonstrates the benefits of GraSS for decompression, offering rapid file reconstruction and competitive performance.

### Comparison of memory and CPU usage performance

**Figure 3:**
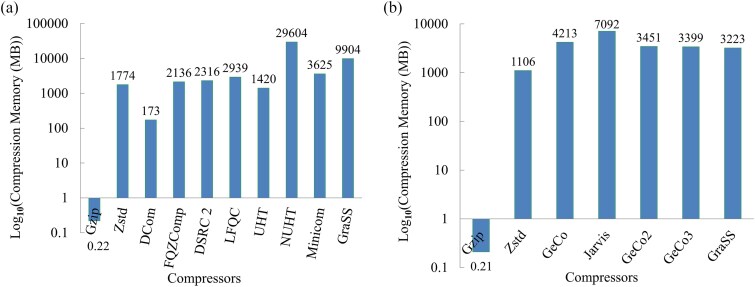
The peak memory use (in MB) of GraSS and (a) nine other state-of-the-art methods was measured for the biggest DNA sequence (GCF_000002235.4) in FASTA format and (b) six other state-of-the-art methods was measured for the largest raw DNA corpus sequence (HoSa).

The proposed method employs a multi-step processing strategy followed by the BSC (Fig. 1) to achieve a higher compression ratio. This results in increased physical memory usage compared to the benchmark algorithms Gzip and Zstd. Among these, Zstd is the second-most efficient performer, while Gzip is the most efficient in terms of memory usage (Fig. 3). General-purpose algorithms consistently consume less memory compared to specialized algorithms because they do not exploit the unique characteristics of genetic sequences. Grammar rule 1 receives the most physical memory allocation in the proposed method, then the replacement model, the BSC compressor, grammar rule 2, sequence extraction, and sequence base frequency computation, in that order. To calculate memory usage, we subtract the amount of free memory from the total memory at the start and end of a specific compressor’s execution. We then calculate the differences between them to derive the outcome.

Figure 3 displays the peak memory consumption of GraSS and nine state-of-the-art methods for ten less-repetitive DNA sequences. For the longest DNA sequence (GCF_000002235.4), Minicom’s peak compression memory is around 3625 MB, which is much larger than its decompression memory of about 990 MB due to the development of a hash table during compression. Using Gzip, the peak compression memory is only approximately 0.22 MB, surpassing other state-of-the-art compression methods. Zstd necessitates approximately 1774 MB, DCom wants around 173 MB, FQZComp utilizes approximately 2136 MB, DSRC 2 consumes about 2316 MB, LFQC occupies roughly 2939 MB, NUHT demands about 29604 MB, and GraSS takes approximately 9904 MB. The memory consumption for the two highly-repetitive RNA sequences can be found in Supplementary File S6. Figure 3 illustrates the maximum memory usage by GraSS and four other advanced algorithms on fifteen raw DNA corpus sequences. When Jarvis processes HoSa, the longest raw sequence in the DNA corpus, it needs a maximum of about 7092 MB of memory. GeCo consumes around 4213 MB of memory; GeCo2 and GeCo3 use approximately the same memory, 3451 MB and 3399 MB, respectively. In contrast, the proposed technique requires approximately 3223 MB of memory.

The initial grammar principle of GraSS may lead to a rise in the number of characters. The compression memory could potentially surpass the peak decompression memory. This occurred with the less repetitive DNA sequence selected, resulting in a compression memory requirement of approximately 9904 MB, which is larger than the decompression memory of around 7539 MB. Supplementary Files S6 and S7 contain detailed information on memory utilization during compression and decompression processes.

The extracted DNA sequences do not contain any special characters [47, 48], while the RNA sequences do not contain any lowercase characters [49]. By adhering to these constraints during the coding process, it is possible to further reduce memory usage.

After each stage, we calculated the CPU utilization of GraSS and other cutting-edge technologies. We derive the final value by averaging three distinct runs. The proposed approach has a lower CPU consumption compared to the advanced GeCo, Jarvis, GeCo2, and GeCo3. During the compression and decompression process, GraSS uses (˜75% and ˜68%), GeCo uses (˜82% and ˜80%), Jarvis uses (˜84% and ˜83%), GeCo2 uses (˜79% and ˜77%), and GeCo3 uses (˜81% and ˜79%), in that order. It may be noted that CPU utilization can exceed 100% because of the multi-core system.

## Conclusion

In this work, we propose a customized reference-free, lossless genome sequence compression method called GraSS. It consists of two distinct phases split into six main steps. In the first phase, it extracts and stores the auxiliary data from a DNA or RNA sequence in raw, FASTA, or multi-FASTA format. Using grammar rules 1 and 2, substitution, and statistical principles followed by a BSC encoder, the raw sequence is compressed in the second phase.

Irrespective of degree of repetitiveness, the proposed algorithm’s performance is very competitive with the state-of-the-art compressors (Tables 1 and 2). For less repetitive DNA sequences, GraSS performs better than the well-known, cutting-edge compressors Gzip, Zstd, DCom, DSRC 2, UHT and NUHT. The proposed approach achieves a WACR of 4.5, outperforming state-of-the-art compressors such as Gzip (3.45), Zstd (4.39), DCom (4.23), DSRC2 (4.21), UHT (3.69), and NUHT (3.97) (Table 1). Even a small improvement over the highly efficient Zstd is noteworthy. Although the LFQC compression ratio (4.88) exceeds that of GraSS, GraSS functions at a speed roughly threefold that of LFQC (Table 1). For highly repetitive RNA sequences, GraSS achieves a WACR of 19.6, which is superior to Gzip (5.65), FQZComp (17.56), DSRC2 (6.63), LFQC (18.23), NUHT (3.98), and Minicom (18.45) (shown in Table 1). This substantial margin demonstrates GraSS’s value in this scenario as well. The WACR of 4.33 for a DNA corpus consisting of fifteen raw sequences is lower than the compression ratios of the most modern and cutting-edge compression algorithms, namely GeCo (4.73), Jarvis (4.78), GeCo2 (4.74), and GeCo3 (4.82), but higher than Gzip (3.69) and Zstd (4.25) (Table 2). The justification is that the proposed approach is primarily designed for the FASTA format, while it can also support raw data. However, these algorithms (GeCo 1, 2, 3, and Jarvis) achieve this at the cost of computational resources.

GraSS performs better than the state-of-the-art compression algorithms Gzip, Zstd, DCom, LFQC, UHT, NUHT, Minicom, Geco, Jarvis, GeCo2, and GeCo3 in terms of compression time, as demonstrated in Tables 1 and 2. Supplementary File S5 provides a record of the duration it took for the decompression process. Undoubtedly, the local execution of GraSS will result in faster performance compared to its remote execution on a cloud server.

The maximal memory use of GraSS (˜3223 MB) is less than that of the most advanced, highly effective algorithms for long-term storage of the DNA corpus, such as GeCo (˜4213 MB), Jarvis (˜7092 MB), GeCo2 (˜3451 MB), and GeCo3 (˜3399 MB). The maximum memory required for the less-repetitive DNA sequence is approximately 172 MB for DCom, 1420 MB for UHT, 29604 MB for NUHT, and 3625 MB for Minicom. About the same amount of memory was needed for FQZComp (˜2136 MB), DSRC 2 (˜2339 MB), and LFQC (˜2909 MB) during compression and decompression. According to SCB [27], Gzip is the most efficient memory performer among functional reference-free compressors. Zstd uses about 1774 MB, which is the second-best usage. For more information, one may refer to Supplementary Files S6 and S7. GraSS uses the least amount of CPU power (˜75%), while the other four (GeCo1, 2, 3 and Jarvis) use around the same amount (˜80%).

GraSS differentiates itself from current genomic sequence compression techniques by eliminating the need for parameter adjustment during operation [27]. It is capable of processing all IUPAC symbols, lowercase letters, identifiers, and line/block lengths. The results (Tables 1 and 2) indicate that GraSS is advantageous for both highly repeated and less repetitive sequences. Moreover, irrespective of the database size, its performance remains consistent. The proposed algorithm’s limitation is that it functions solely as a reference-free method, utilizes a single backend compressor (BSC), and is implemented in Java; however, a C/C++ implementation could enhance the algorithm’s performance to a degree.

There is still significant room for improvement. Palindromes, other repeating structures within a sequence, and precise or approximate repeats can all contribute to increasing the compression ratio of highly repetitive genomic sequences. Additionally, applying disk write optimization techniques to support frequent access can significantly reduce decompression time. Finally, GraSS can be employed with other formats (such as FASTQ files) and datasets (such as protein datasets). Any sequence analysis approach that works with raw data can also be used with compressed data, typically at the expense of accuracy.

**Key Points**

- The article discusses a lossless compression method for genomic sequences without references using grammar, statistics, and substitution (GraSS) rules.
- The article proposes an algorithm, GraSS, comprising six major steps divided into two phases, implemented using Java on the Amazon Web Services (AWS) Linux platform.
- The article showcases the compression method for wide-scale, less-repetitive, or highly-repetitive sequences, and all IUPAC codes enable compression in the raw, FASTA-ALL (FASTA), or Multi-FASTA formats.
- The article considers identifiers, line and block lengths, and small cases that may arise in a sequence.
- The article highlights that the proposed method does not depend on parameter settings because an algorithm involving parameter settings becomes complex and time-consuming.

## Supplementary Material

Supplementary_File_S1_elae050

Supplementary_File_S2_elae050

Supplementary_File_S3_elae050

Supplementary_File_S4_elae050

Supplementary_File_S5_elae050

Supplementary_File_S6_elae050

Supplementary_File_S7_elae050

## Code availability

The study’s source codes, test data samples, and instructions are all freely accessible at https://github.com/AnshuOishik/GraSS. You can use them for free for private, non-commercial
